# Diets Differently Affect Bone Health: Murine Models

**DOI:** 10.3390/ijms27146094

**Published:** 2026-07-08

**Authors:** Donatella Mentino, Alessia Annicchiarico, Alessia Provera, Alessandro Antonioli, Vesa-Matti Leino, Salvatore Sutti, Flavia Prodam, Heikki Suhonen, Grazia Paola Nicchia, Maria Mastrodonato, Maria Felicia Faienza, Giacomina Brunetti

**Affiliations:** 1 Department of Biosciences, Biotechnologies and Environment, University of Bari Aldo Moro, 70125 Bari, Italy; donatella.mentino@uniba.it (D.M.); alessia.annicchiarico@uniba.it (A.A.); graziapaola.nicchia@uniba.it (G.P.N.); maria.mastrodonato@uniba.it (M.M.); 2 Department of Health Science, University of Piemonte Orientale, 28100 Novara, Italy; alessia.provera@uniupo.it (A.P.); alessandro.antonioli@uniupo.it (A.A.); salvatore.sutti@uniupo.it (S.S.); flavia.prodam@uniupo.it (F.P.); 3 X-Ray Laboratory, Department of Physics, University of Helsinki, P.O. Box 64, FI-00014 Helsinki, Finland; vesa-matti.leino@helsinki.fi (V.-M.L.); heikki.suhonen@helsinki.fi (H.S.); 4 Pediatric Unit, Department of Precision and Regenerative Medicine and Ionian Area, University of Bari, Aldo Moro, Piazza G. Cesare 11, 70124 Bari, Italy; mariafelicia.faienza@uniba.it

**Keywords:** diet, bone health, mouse model, bone cells

## Abstract

Bone is a dynamic specialized connective tissue that undergoes continuous remodeling to preserve its health. Bone health is influenced throughout life by a combination of genetic, hormonal, and environmental factors, as well as physical activity and diet. This study aims to evaluate the effects of diets with different fat content on the femurs of mice fed for 16 or 20 weeks on a normal diet (ND16w and ND20w) or a Western diet (WD16w and WD20w) and for 20 weeks with their combinations on a ketogenic diet (KD) (WD + ND20w, ND + KD20w, and WD + KD20w). Micro-CT analysis on femoral cancellous bone revealed a non-significant trend toward decreased bone volume fraction (BV/TV) and trabecular thickness in mice fed a combined WD + KD20w compared to WD20w. Cortical bone thickness was significantly lower in mice fed WD16w and WD20w compared to those fed ND16w and ND20w (*p* = 0.049 and *p* = 0.039, respectively), in mice fed WD20w Ct.Th increased compared to WD + ND20w (*p* = 0.024) and a strong decrease is evident when comparing WD + ND20w to WD + KD20w (*p* < 0.0004). Consistently, histological analysis revealed that the number of osteoclasts per bone perimeter on cancellous bone increases compared with ND20w with ND + KD20w (*p* = 0.007) and WD + ND20w with ND + KD20w (*p* = 0.0006). In addition, a decrease in osteoblasts was observed (*p* < 0.041) in cortical bone, comparing ND20w with ND + KD20w; this suggests that the KD may have differential effects depending on the baseline condition. Osteocyte numbers did not significantly change when comparing the different treatments. Masson staining supports micro-CT results on both cortical and cancellous bone. In conclusion, transition from a high-fat diet to a normal diet may partially restore cortical bone health, whereas transition to a ketogenic diet exerts a trend toward additional detrimental effects on trabecular bone.

## 1. Introduction

Bone is a dynamic tissue that undergoes continuous remodeling to preserve its health, shape, and structural integrity while regulating mineral homeostasis [1,2]. Bone remodeling is a complex biological process that involves the resorption activity of mineralized bone tissue by osteoclasts (OCs) and the production of new bone matrix by osteoblasts (OBs) [3]. It is orchestrated by osteocytes originating from osteoblasts that become embedded in the bone matrix over time. These cells form an extensive network by connecting through long cellular processes within tiny canaliculi. Making up about 90% of bone cells, osteocytes play a crucial role in communicating with surface bone cells and regulating bone remodeling [4]. Together, these processes maintain the dynamic balance of bone renewal and repair, ensuring skeletal health and adaptability over time [5].

Bone health is influenced throughout life [6,7] by a combination of genetic, hormonal, and environmental factors, in particular physical activity and nutrition/diet [8,9]. Calcium and vitamin D intake are the critical nutritional factors most studied until now. Diets with changes in their carbohydrate, protein and fat content impact differently on bone health; in particular, high-fat content is associated with bone loss [10]. Consistently, using different mouse models and diets, researchers have investigated their impact on bone health, showing interesting results for Western and ketogenic diets (WD and KD, respectively). Mimicking a Western fast-food diet with a high content of fats and carbohydrates, the WD may have a particularly harmful impact, as it not only contains high levels of saturated fats, processed and simple carbohydrates but is also deficient in calcium and other essential minerals that support calcium absorption and bone health [11,12]. WD showed strong effects on skeletal health as demonstrated in animal models [10]. In detail, in 9-week-old female C57bL/6J mice after 10 weeks of WD, the tibial cross-sectional area, cortical thickness, and maximum load were significantly decreased, whereas the serum levels of the OC marker Tartrate-Resistant Alkaline Phosphatase (TRAP) and the pro-osteoclastogenic Receptor activator of nuclear factor kappa-B ligand (RANKL) were increased [13]. In ovariectomized rats, WD significantly decreased bone mineral density (BMD) in the tibia and femora, serum osteocalcin (OCN) levels, urine deoxypyridinoline (DPD) amount, as well as the OC-specific marker expression cathepsin-K, suggesting that WD affected bone health [14]. Other studies have demonstrated the detrimental effects of WD on tibial cortical bone [15]. Different studies have reported that the bone loss associated with a WD may be due to the augmented levels of inflammatory cytokines with consequent increased OC activity [13].

The KD represents an extremely high-fat diet (HFD) affecting negatively bone microstructure [16]. KDs are also associated with potential negative effects on bone health. Previous research has highlighted the impact of KDs on bone mineral content (BMC), osteopenia, osteoporosis, hypercalciuria, urine acidification, hypocitraturia, and the risk of decreased BMD [16,17,18,19]. In detail, Wu et al. [17] used micro-CT in 8-week-old mice for 12 weeks and showed that both femoral cancellous and cortical bone were negatively affected. Aikawa et al. [20] fed KD-aged mice under exercise training, showing impaired bone mass, cancellous microstructure, and compromised beneficial effects of physical activity on bone health. Liu et al. [21] reported that KD delays spinal fusion in rats after surgery. Xu et al. [22] reported that rats fed with KD showed high TRAP activity and reduced alkaline phosphatase activity.

Based on this background, we aimed to evaluate the effects of different dietary patterns on bone health in mouse models. Thus, we fed mice for 16 and 20 weeks with a normal diet (ND16w, ND20w) or Western diet (WD16w, WD20w), and for 20 weeks with combinations of normal diet + Western diet (ND + WD20w), normal diet + ketogenic diet (ND + KD20w) or Western diet + ketogenic diet (WD + KD20w).

## 2. Results

### 2.1. Diet Effects on Bone Microstructures

**Figure 1:**
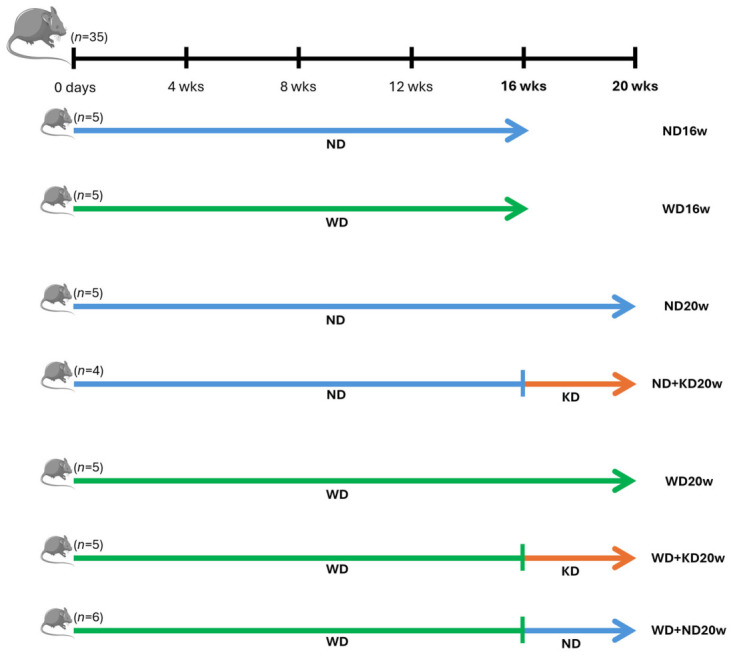
Experimental design. 35 mice were divided randomly into seven groups: *n* = 5 mice fed with normal diet for 16 weeks (ND16w); *n* = 5 fed with Western diet for 16 weeks (WD16w); *n* = 5 mice fed with normal diet for 20 weeks (ND20w); *n* = 4 mice fed with normal diet for 16 weeks and then with Ketogenic diet for 4 weeks (ND + KD20w); *n* = 5 fed with Western diet for 20 weeks (WD20w); *n* = 5 mice fed with Western diet for 16 weeks and then with Ketogenic diet for 4 weeks (WD + KD20w); *n* = 6 mice fed with Western diet for 16 weeks and then with normal diet for 4 weeks (WD + ND20w).

Thirty-five 4-week-old male mice, strain (C57BL/6J), were purchased, and after 4 weeks of acclimatization, 8-week-old mice were fed for 16 and 20 weeks with a normal diet (ND16w, ND20w) or a Western diet (WD16w, WD20w), and for 20 weeks with combinations of normal diet + Western diet (ND + WD20w: 16 weeks ND + 4 weeks WD), normal diet + ketogenic diet (ND + KD20w: 16 weeks ND + 4 weeks KD), or Western diet + ketogenic diet (WD + KD20w: 16 week WD + 4 week KD20) (Figure 1).

**Figure 2:**
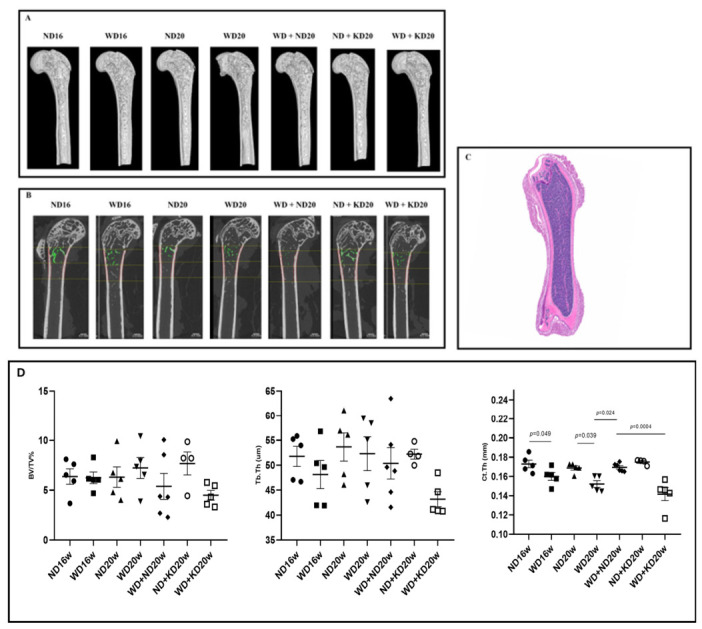
Effect of diets on bone microstructure by micro-CT. 3D and 2D micro-CT reconstruction images of distal femurs of mice (**A**,**B**). Red and green colors indicate the measured cortical and trabecular regions, respectively scale bar corresponds to 0.5 mm (**B**). Typical hematoxylin-Eosin staining of a representative femur, magnification 4× (**C**). Graphs reporting BV/TV%, Tb.Th and Ct.Th (**D**). Data are shown as Mean ± SEM.

In the present study, utilizing micro-CT, we analyzed the femur microarchitectural features from C57BL/6J mice fed ND, WD, KD, and/or their combination for 16 and 20 weeks (Figure 2, Table 1). Micro-CT analysis of the cancellous bone region of femurs showed that BV/TV%, Tb.Th, Tb.N, and Tb.Sp did not display significant variation between the different kinds of diets, although a trend to a decrease was evident for BV/TV% and Tb.Th comparing WD20 with WD + KD20w. In cortical bone, a significant decrease was observed in Ct.Th comparing mice fed ND16w or ND20w with those fed WD16w or WD20w (*p* = 0.049 and *p* = 0.039, respectively). Interestingly, mice fed the combined WD + ND20w showed a significant increase in Ct.Th compared to mice fed WD20w, *p* = 0.024. Furthermore, mice fed the combined WD + KD20w diet showed a significant decrease in Ct.Th with respect to mice fed the combined WD + ND20w diet (*p* = 0.0004). All these data highlight the detrimental effects of KD on cortical bone, whereas WD negatively affects cortical bone compared with ND both at 16 and 20 weeks of treatment.

### 2.2. Effect of Diets on Osteoclast Number Ex Vivo

**Figure 3:**
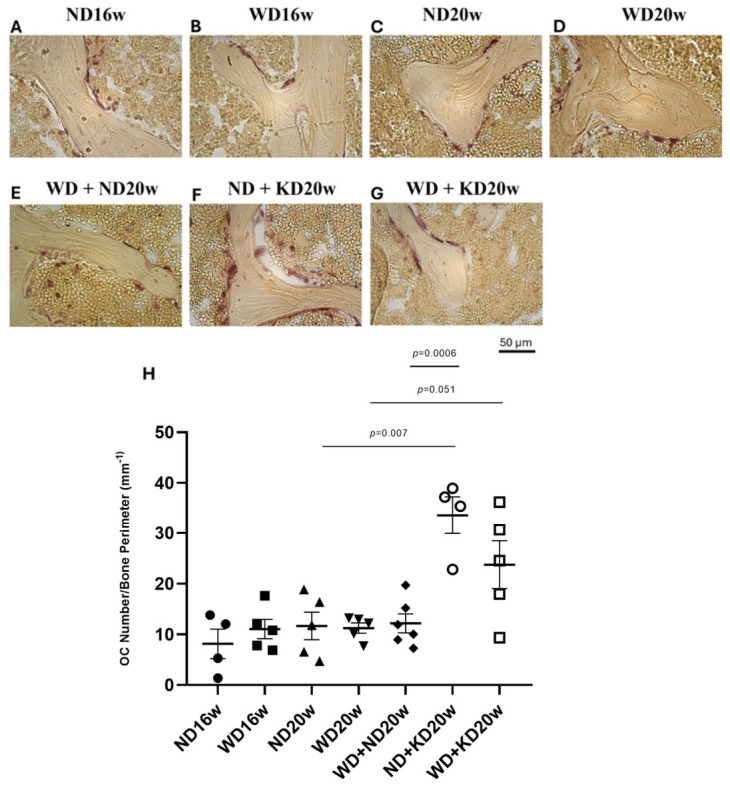
Effect of diets on the OC number in the femur bone. Representative images of TRAP-stained osteoclasts in femur sections from ND16w (**A**), WD16w (**B**), ND20w (**C**), WD20w (**D**), WD + ND20w (**E**), ND + KD20w (**F**), and WD + KD20w (**G**) mice, together with osteoclast counts per bone perimeter (**H**) in femoral trabecular sections from all mice. Data are shown as Mean ± SEM. Magnification ×40.

The interesting findings derived from micro-CT analysis prompted us to count bone cells through histological analysis in cortical and trabecular bone. Histomorphology of trabecular bone using TRAP staining to highlight OCs was performed, and the number of OCs per trabecular bone perimeter N.Oc/Tb.Pm was assessed (Figure 3). Interestingly, the number of OCs strongly increased in WD + KD20w mice compared with the ND20w group, *p* = 0.007. Furthermore, mice fed WD + KD20w with respect to the WD20w group showed a trend to enhancement, but it was not statistically significant *p* = 0.051. Of note, the trabecular bone of ND + KD20w mice displayed a significantly higher number of OCs compared to the WD + ND20w group (*p* = 0.0006). These findings support the micro-CT results and indicate that the combination of the KD diet with ND or WD over 20 weeks led to a marked increase in OC numbers, suggesting enhanced bone resorption under these conditions.

### 2.3. Effect of Diets on Osteoblast Number Ex Vivo

**Figure 4:**
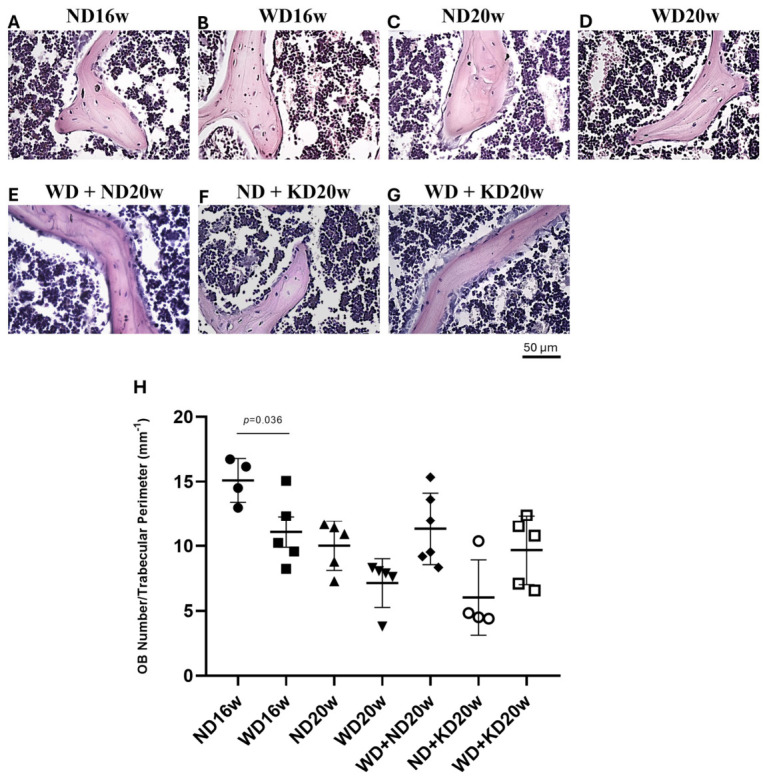
Effect of diets on the number of OB in the femur bone. Representative images of Hematoxylin-Eosin (H&E)-stained osteoblasts in femur sections from ND16w (**A**), WD16w (**B**), ND20w (**C**), WD20w (**D**), WD + ND20w (**E**), ND + KD20w (**F**), and WD + KD20w (**G**) mice, together with osteoblast counts per bone perimeter (**H**) in femoral trabecular sections from all mice. Data are shown as Mean ± SEM. Magnification ×40.

The H&E-staining analysis of the number of OB per bone trabecular perimeter (N.Ob/Tb.Pm) in the femoral cancellous bone region showed that mice fed for 16 weeks with WD had a reduction compared to ND, *p* = 0.036; Figure 4. The N.Ob/Tb.Pm showed a trend to a decrease comparing ND + KD20w versus ND20w, as well as ND + KD20w versus WD + ND20w, without reaching statistical significance. An increase in OB number was observed in mice fed WD + ND20w combined diet versus WD20w, but not significant.

**Figure 5:**
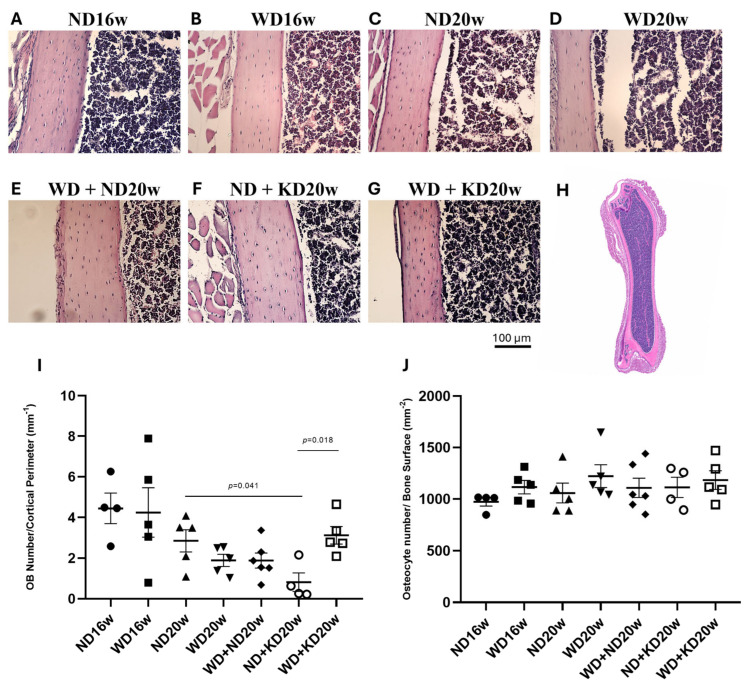
Effect of diets on OB and osteocyte number in the femur cortical bone. Representative images of Hematoxylin-Eosin (H&E)-stained osteoblasts and osteocytes in femur section from ND16w (**A**), WD16w (**B**), ND20w (**C**), WD20w (**D**), WD + ND20w (**E**), ND + KD20w (**F**), and WD + KD20w (**G**) mice, together with osteoblast counts per bone perimeter (**I**), and osteocyte counts per bone surface (**J**) in femoral trabecular sections from all mice. Data are shown as Mean ± SEM. Magnification ×20. A representative femur with 4× magnification is also reported (**H**).

Furthermore, a significant decrease in the OB number per bone cortical perimeter (N.Ob/Ct.Pm) was observed in mice fed the combined ND + KD20w diet compared to ND20w (*p* = 0.041), whereas an increase was observed comparing WD + KD20w with WD20w (*p* = 0.018). This suggests that the KD may have differential effects depending on the baseline condition (Figure 5).

### 2.4. Diets and Osteocyte Number Ex Vivo

The results about the strong effects of diet on cortical bone prompted us to evaluate osteocytes. As shown in Figure 5E,H.J, staining revealed that osteocyte number per bone surface (Ot.N/BS) did not significantly change when comparing the different conditions, although a trend toward an increase is evident comparing the group of mice fed with the WD16w or WD20w vs. the groups fed with the ND16w or ND20w, respectively, and ND + KD20w vs. ND20w fed mice; while the group fed with WD + KD20w showed a non-significant decrease compared to WD20w; Figure 5A–G.

### 2.5. Effect of Diets on Osteoid Surface

**Figure 6:**
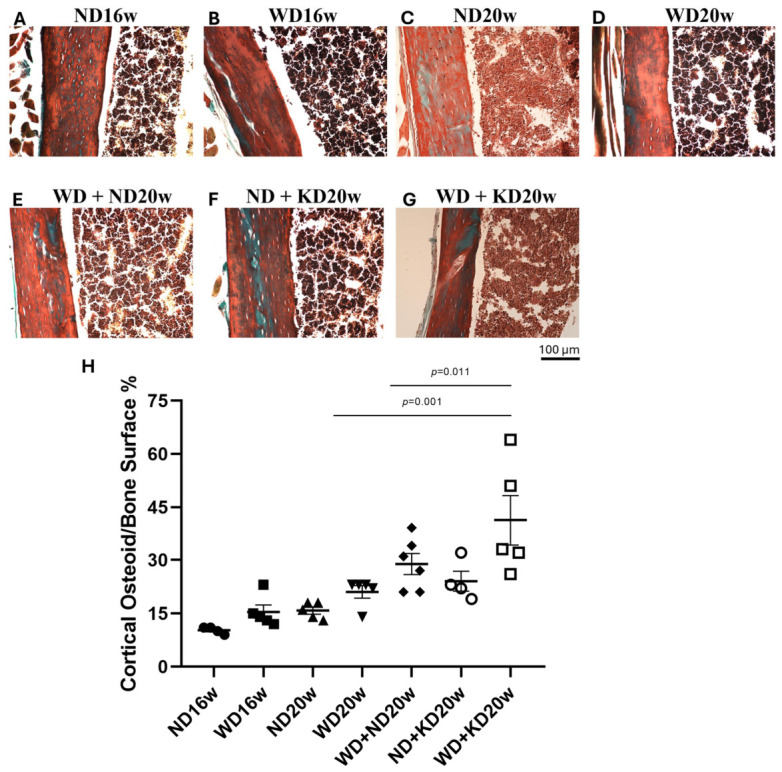
Effect of diets on the osteoid surface of cortical bone. Representative images of Masson trichrome Goldner-stained sections of cortical sections of femurs from ND16w (**A**), WD16w (**B**), ND20w (**C**), WD20w (**D**), WD + ND20w (**E**), ND + KD20w (**F**), and WD + KD20w (**G**) mice, together with a graph reporting the evaluation of osteoid on bone surface (**H**). Data are shown as Mean ± SEM. Magnification ×20.

**Figure 7:**
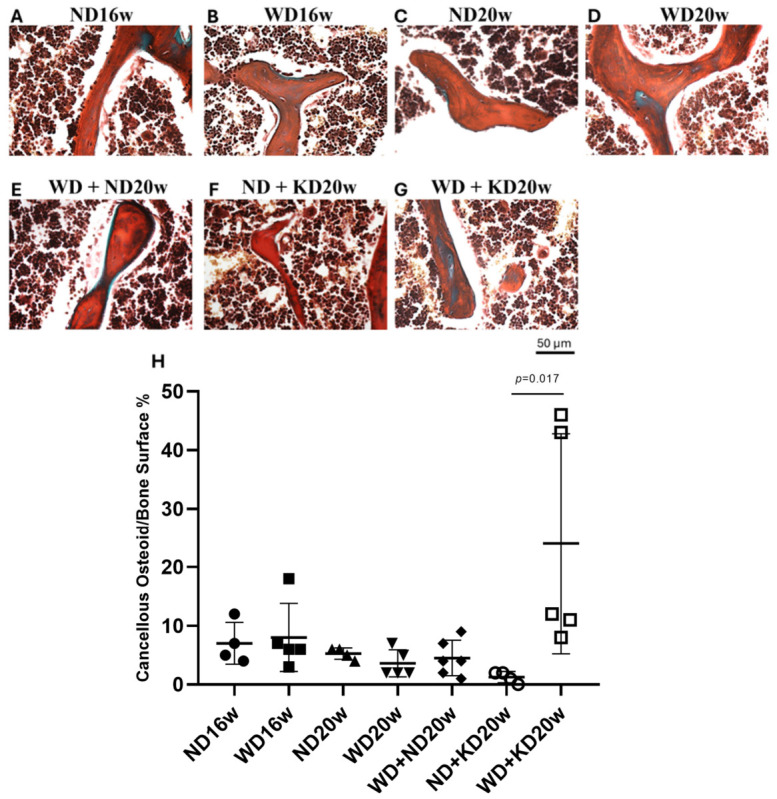
Effect of diets on the osteoid surface of trabecular bone. Representative images of Masson trichrome Goldner-stained sections of trabecular sections of femurs from ND16w (**A**), WD16w (**B**), ND20w (**C**), WD20w (**D**), WD + ND20w (**E**), ND + KD20w (**F**), and WD + KD20w (**G**) mice, together with a graph reporting the evaluation of osteoid on bone surface (**H**). Data are shown as Mean ± SEM. Magnification ×40.

The obtained results prompted us to evaluate the osteoid surface in both cortical and cancellous bone (Figure 6 and Figure 7). Masson staining evaluations supported micro-CT results, showing an increase in the green area (osteoid surface)/bone surface percentage in cortical bone with the increase of fat in the diet. In detail, a significant increase was evident when comparing WD + KD20 vs. WD20w (*p* = 0.011) and vs. ND20w (*p* = 0.001); Figure 6. The highest area of osteoid surface was measured in the WD + KD20 group (2-fold compared with the WD20w group); Figure 6.

Cancellous bone evaluation showed a significant increase in osteoid surface compared to the WD + KD20w group vs. the ND + KD20w (*p* = 0.017); Figure 7.

## 3. Discussion

In this work, we examine the effects of different dietary patterns, ND, WD, and KD, with progressively increasing fat content, ranging from 13% to 90.5%, on bone health in mouse models. We used micro-CT analysis to demonstrate that a significant decrease was observed in Ct.Th in cortical bone in mice fed with WD16w and WD20w compared to ND16w and ND20w, respectively, as well as mice fed the combined WD + KD20w diet vs. WD + ND20w diet. Cancellous BV/TV% and Tb.Th showed a non-significant decreasing trend in combined WD + KD20w diet compared to mice fed only WD20w. Consistently, significant differences emerged from OC, OB, and osteocyte counting, as well as Mallory staining in our samples.

Compared to studies already carried out to investigate the effects of WD and KD on bone health feeding animals for different periods, the novelty of our study lies in evaluating the impact of WD for a long time (20 weeks) and the switching from WD16w to either ND or KD for 4 weeks as well as from ND16w to KD for 4 weeks, specifically WD + ND20w, WD + KD20w, and ND + KD20w. In detail, we found a significant reduction in femur Ct.Th caused by WD at both times of 16 and 20 weeks over ND. Previous authors evaluated the impact of WD in Sprangue-Dewley female rats for 10 [15,23] and for 12 weeks [14] and reported that WD negatively affects bone health. Hou et al. [23] showed that WD decreased the relative size of the femoral neck cortical shell and increased the trabecular core compared to rats fed a control diet. Li et al. [15] found that WD altered bone mechanical properties; in fact, the tibias had significantly smaller maximum load, failure energy, and tensile stress at the proportional limit than controls. Furthermore, Dong et al. [14] reported detrimental effects of WD on cancellous bone with reduced Tb.BMD in the tibia head and femoral end with respect to ND fed rats. Interestingly, Lorincz et al. [13], using female mice fed for 20 weeks with WD, demonstrated a significant reduction of Ct.Th, cross-sectional area, and at maximum load/load at maximum in the tibia.

In line with these findings, we also report a significant decrease in OB numbers observed in WD16w compared to ND16w in cancellous bone, suggesting that WD may have a negative effect on OB maintenance or proliferation. This could be due to the high-fat content and inflammatory nature of the WD, which might impair OB cellular functions. Additionally, the OB number in cortical bone decreased compared with ND20w with ND + KD20w, suggesting the key role of a normal diet for bone health. Furthermore, the OB number increased in the comparison of ND + KD20w to WD + KD20w, suggesting that shifting from WD to ND (dietary intervention) may balance some of the negative effects associated with WD consumption, and the effect of KD may have exerted a major detrimental effect with respect to the switch linked to WD. The literature data report a significant decrease in OBs in HFD mice compared to ND, and serum procollagen type I N-pro-peptide (PINP) levels were consistently reduced in the same groups [24]. Interestingly, in vitro experiments have demonstrated that treating the osteoblastic cell line MC3T3 with NEFA (the fatty acids oleate and palmitate, in a 1:2 mixture) impairs osteoblastogenesis, as demonstrated by the reduction in RUNX2 levels [25].

Notably, we found that switching from WD to KD resulted in a non-significant decrease in BV/TV% compared to controls, along with Tb.Th, implying a negative effect of either WD or KD on the quality of trabecular in mice. Ct.Th was significantly affected in all treatments.

In the same vein, Wu et al. [17] and Liu et al. [18] demonstrated that mice fed a KD for 12 weeks exhibited compromised micro-architecture of cancellous bone and the morphological structure of the cortical femur, together with altered mechanical properties. These macroscopic features are linked to an increase in the number of OCs [17,18] and a decrease in osteocalcin-positive cells [18]. Increased TRAP and cathepsin-K mRNA levels were also detected in HFD [24]. Consistently, our histological analysis revealed that the number of OCs on cancellous bone strongly increased in WD + KD20w compared with WD + ND20w and WD + ND20W compared with ND + KD20w. However, in cortical bone, an increase was observed in the number of OBs compared with the WD + KD20w diet with WD20w, suggesting that KD may have differential effects depending on the baseline condition. The literature data link the general increase in osteoclastogenesis linked to the HFD with enhanced levels of the pro-osteoclastogenic cytokine RANKL and the RANKL/OPG ratio [25].

It has been reported that RANKL can be produced by OBs and adipocytes [26]. In fact, an increased number of marrow adipocytes is linked to HFD. At the cellular level, adipocytes and OBs originate from Bone Marrow Stromal cells; obesity promotes adipocyte differentiation through the PPARγ pathway and inhibits OB differentiation and thus bone formation [27]. Consistently, fat accumulation in the bone marrow stimulates adipogenesis [28], leading to increased production of the pro-inflammatory cytokines by the bone marrow that promote the proliferation and differentiation of OCs, cells responsible for bone resorption. Inflammation leads to increased osteoclastogenesis, which correlates with increased RANKL and decreased OPG. This suggests that HFD may contribute to bone loss by modulating the RANK/RANKL/OPG axis in favor of osteoclastogenesis [29], resulting in enhanced OC activity and reduced OB turnover [28].

Interestingly, we did not find significant differences in osteocyte counts associated with the different diets. Our data were supported by the literature data that report non-significant variation in osteocyte number and markers in HFD [30,31,32]; however, accelerated osteocyte senescence was evident, as demonstrated by high expression levels of p16 and p21 [30]. Dole et al. reported that WD increased perilacunar/canalicular remodeling through osteocyte-intrinsic regulation of enzymes implicated in this signaling, compromising lacuna-canalicular network integrity and mitochondrial activity, and inducing cellular senescence [32].

In general, consumption of diets composed of high amounts of saturated fat has been reported to lead to the formation of insoluble soap complexes, preventing calcium absorption [13]. Furthermore, it is known that ingestion of sucrose alone determines calcium excretion in the urine or hypercalciuria. Consequently, diets that include both high-saturated fat/sucrose (i.e., our WD) have a profound effect on skeletal structural integrity, also due to the effect on calcium absorption and excretion [13].

Thus, with high fat, the increase in proinflammatory cytokines, the combined effect of the common precursors shared by osteoblasts and adipocytes, reduced osteoblastogenesis, the altered RANKL/RANK/OPG axis favoring osteoclastogenesis, the senescence of osteocytes, and altered calcium absorption, together generate detrimental effects on bone.

One limitation of our study is that we did not evaluate serum biochemical markers, such as calcium, PTH, vitamin D, bone resorption marker CTX-1, formation marker P1NP, glucose, insulin, leptin, and RANKL/OPG.

In human studies, it is well known that WD, and thus obesity, is linked to osteopenia/osteoporosis. However, different authors also focused on KD and bone effects, with contrasting results depending on K calory/Kcal content, age, gender, and the presence of a physical activity protocol. Carter et al., after three months of treatment with KD, detected no significant changes in bone serum markers [33]. Athletes after 3.5 weeks of KD showed an increase in bone resorption markers [34]. In an additional study, healthy women following an 8-week KD reported an increase in BMD compared with another female group fed a normal diet [35].

## 4. Materials and Methods

### 4.1. Mouse Models and Experiment Design

This animal interventional study is in accordance with the European Law Implementation of Directive 2010/63/EU, and all experimental protocols were reviewed and approved by the Veterinary Department of the Italian Ministry of Health (authorization No. 411/2020-PR, 5 May 2020).

Thirty-five 4-week-old male mice, strain (C57BL/6J), were purchased from Charles River Laboratories Italy (Calco, LC, Italy). After four weeks of acclimatization, 8-week-old mice were fed for 16 and 20 weeks with a normal diet (ND16w, ND20w) or a Western diet (WD16w, WD20w), and for 20 weeks with combinations of normal diet + Western diet (ND + WD20w: 16 weeks ND + 4 weeks WD), normal diet + ketogenic diet (ND + KD20w: 16 weeks ND + 4 weeks KD), or Western diet + ketogenic diet (WD + KD20w: 16 week WD + 4 week KD20), (Figure 1).

Diet composition was the following:

- ND (Control group): mice were fed a standard rodent diet consisting of 13% Kcal fat, 20% Kcal proteins, and 67% Kcal carbohydrates (Envigo, Bresso, Mi, Italy).
- WD: mice were fed a diet consisting of 42% Kcal fat, 15% Kcal proteins, 43% Kcal carbohydrates, and enriched with 1.25% cholesterol (Laboratorio Dottori Piccioni, Gessate, Italy).
- KD: mice were fed a choline-sufficient, cholesterol-free diet consisting of 90.5% Kcal vegetal fat (hydrogenated coconut oil), 9.2% Kcal proteins, and 0.3% Kcal carbohydrates (Laboratorio Dottori Piccioni, Gessate, Italy).

Animals had free access to the diet and tap water ad libitum, were housed 3–5 per cage and were exposed to a 12-h light/12-h dark cycle. After 16 and 20 weeks of diet, animals were euthanized by isoflurane and cervical dislocation. Femurs were rapidly removed, fixed with 4% (vol/vol) formaldehyde for 24 h at 4 °C, and processed for microcomputed tomography (Micro-CT) and histological analysis.

### 4.2. Microcomputed Tomography Analysis of Femurs

For micro-CT, images were acquired using Phoenix Nanotom S (Waygate Technologies, Huerth, Germany). The bone was mounted so that its longitudinal axis was approximately on and parallel to the tomographic rotation axis. The following settings were used for tomographic scans: X-ray tube voltage 60.00 kV; tube current 120.00 μA; integration time 5 × 250.00 ms/projection; number of projections 800 over 360° rotation; and magnification 20, voxel size 5 × 5 × 5 μm^3^. The resulting field of view of the reconstructed image was approximately 5 × 5 × 5 mm^3^. In order to scan a sufficiently long part of the sample along the bone axis, two scans of each sample were made by moving the sample stage vertically by 4 mm between scans. The image reconstruction was performed using the Phoenix Datos|x 2 rec (version 2.4.0) software package that was included with the scanner. The two imaged sections of the sample were reconstructed separately and then joined together by a custom ImageJ (version 1.53t) script that optimizes the join position based on the image data to account for motor movement inaccuracies.

The reconstructed 3D images were then all processed through the exact same image-processing and measurement workflow, in which the ImageJ software and its plugins MorphoLibJ (version 1.6.0) and BoneJ (version 7.0.17) were used [36,37]. Segmentation of the 3D images was performed (in 2D) using the image processing method of morphological filtering on binary images, which were obtained by thresholding the original images with Otsu’s thresholding algorithm. The algorithm utilizes the image histogram to calculate a level of brightness that determines whether an individual voxel (3D pixel) is turned white (if its brightness is above the level) or black (if it is below). Thresholding the images delineates the edge of the bone-density material quite precisely.

The segmentation workflow consisted of a sequence of image-processing operations that were performed consecutively on the results of previous operations in the workflow. The primary image processing method used in the workflow was morphological filtering, which enables the segmentation of trabecular and cortical bone based on their morphology. The segmentation workflow that was used is similar to the workflow introduced by Herbst et al. [38].

The cortical parameters measured included cortical thickness (Ct.Th), total cross-sectional area (Tt.Area), cortical bone perimeter (Ct.Pm), and marrow cross-sectional area (Marrow Area). The trabecular parameters measured included bone volume/total volume (BV/TV), number (Tb.N), thickness (Tb.Th), and separation (Tb.Sp).

The areas and the perimeter (Tt.Area, Marrow Area, and Ct.Pm), as well as the volumes for BV/TV, were measured with standard ImageJ, whereas the thicknesses and the separation (Ct.Th, Tb.Th, and Tb.Sp) were measured with the ImageJ-plugin BoneJ. Tb.N was calculated as the inverse of the sum of Tb.Th and Tb.Sp. All means and standard deviations were calculated by ImageJ and BoneJ.

The measurement region was chosen to start at the level where the curvilinear epiphyseal plate reaches its furthest extent toward the diaphysis. This reference point could be similarly found on all the samples. The measurement region was chosen to continue towards the diaphysis for a distance of 1.2 mm for trabecular bone and 2.4 mm for cortical bone.

### 4.3. Histological Analysis

To evaluate bone health and perform bone histomorphometry, mouse femurs were decalcified and embedded in paraffin wax. Serial sections, 5 µm thick, were cut using a rotary microtome (Leica RM 2155, Leica, Wetzlar, Germany) [39,40]. The sections were deparaffinized, rehydrated through a graded alcohol series, and stained as follows:

Hematoxylin and Eosin (H&E, Sigma Aldrich, Milan, Italy) [41] staining was applied for OB analysis, assessing OB number per bone cancellous perimeter (N.Ob/Tb.Pm) and per bone cortical perimeter (N.Ob/Ct.Pm). The osteocyte number was determined on the same sections.

Tartrate-Resistant Alkaline Phosphatase (TRAP, Sigma Aldrich, Milan, Italy) staining solution was used to evaluate OC number per bone cancellous perimeter (N.Oc/Tb.Pm). Images were captured using a Nikon Eclipse E600 light microscope equipped with a DS-Fi3 microscope camera (Nikon Instruments Ltd., Campi Bisenzio, Florence, Italy). Masson trichrome Goldner staining was performed using a commercially available kit, according to the manufacturer’s instructions (Masson trichrome Goldner Bio-Optica, Milan, Italy).

All measurements were performed using ImageJ; Adobe Photoshop 2024 (version 25.12.1) was used for Masson evaluation.

### 4.4. Statistical Analysis

Statistical analyses were conducted using one-way ANOVA and post-hoc test Tukey, according to the Statistical Package for the Social Sciences software (IBM SPSS, Armonk, NY, USA), version 25. Data were reported as Mean ± medium standard error (SEM). Thirty-five mice were used for the micro-CT analysis ND16w (n = 5), WD16 (n = 5), ND20 (n = 5), WD20 (n = 5), WD + ND20w (n = 6), ND + KD20w (n = 4), and WD + KD20w (n = 5). Thirty-four mice were used for the histological analysis ND16w (n = 4), WD16 (n = 5), ND20 (n = 5), WD20 (n = 5), WD + ND20w (n = 6), ND + KD20w (n = 4), and WD + KD20w (n = 5). The results were considered statistically significant at *p* < 0.05. GraphPad was used for graphs.

## 5. Conclusions

Transition from a high-fat diet to a normal diet may partially restore cortical bone health, whereas a transition to a ketogenic diet shows a trend to exert additional detrimental effects on trabecular bone.

## Figures and Tables

**Table 1:** Micro-CT parameters.

	BV/TV%	Tb.N [1/mm]	Tb.Th µm	Tb.Sp µm	Ct.Th mm	Tt.Area mm^2^	Ct.Pm mm	Ma.Ar mm^2^
**ND16**	6.36 ± 1.73	2.75 ± 0.25	51.77 ± 4.51	313.93 ± 27.98	0.1729 ± 0.0087 vs. WD16 ***p* = 0.049**	2.67 ± 0.18	6.66 ± 0.27	1.76 ± 0.13
**WD16**	6.24 ± 1.29	3.06 ± 0.35	48.14 ± 6.30	282.07 ± 31.80	0.1600 ± 0.0090	2.77 ± 0.35	6.77 ± 0.53	1.91 ± 0.26
**ND20**	6.31 ± 2.28	2.66 ± 0.19	53.67 ± 6.32	323.53 ± 21.32	0.1688 ± 0.0051 vs. WD20 ***p* = 0.039**	2.65 ± 0.28	6.69 ± 0.39	1.76 ± 0.21
**WD20**	7.23 ± 2.36	2.88 ± 0.47	52.31 ± 7.59	304.07 ± 62.11	0.1521 ± 0.0078	2.91 ± 0.39	6.96 ± 0.45	2.07 ± 0.32
**WD + ND20**	5.38 ± 3.19	2.80 ± 0.25	50.37 ± 7.73	309.63 ± 28.98	0.1695 ± 0.0042 vs. WD20 ***p* = 0.024**	2.76 ± 0.47	6.73 ± 0.62	1.86 ± 0.39
**ND + KD20**	7.68 ± 2.30	2.84 ± 0.08	52.21 ± 1.98	300.45 ± 10.69	0.1750 ± 0.0028	2.81 ± 0.21	6.82 ± 0.30	1.87 ± 0.18
**WD + KD20**	4.49 ± 1.09	3.01 ± 0.14	43.19 ± 3.37	289.53 ± 12.59	0.1417 ± 0.0151 vs. WD + ND 20 ***p* = 0.0004**	2.58 ± 0.30	6.52 ± 0.43	1.85 ± 0.29

Bone volume/total volume (BV/TV), trabecular number (Tb.N), thickness (Tb.Th), and separation (Tb.Sp), cortical thickness (Ct.Th), total cross-sectional area (Tt.Area), cortical bone perimeter (Ct.Pm), and marrow cross-sectional area (Marrow Area, Ma.Ar).

## Data Availability

The raw data supporting the conclusions of this article will be made available by the authors on request.
